# Quality of life and subjective symptom impact in Japanese patients with myotonic dystrophy type 1

**DOI:** 10.1186/s12883-022-02581-w

**Published:** 2022-02-14

**Authors:** Haruo Fujino, Toshio Saito, Masanori P. Takahashi, Hiroto Takada, Takahiro Nakayama, Osamu Imura, Tsuyoshi Matsumura

**Affiliations:** 1 Department of Child Development, United Graduate School of Child Development, Osaka University, 2-2 Yamadaoka, Suita, 5650871 Japan; 2 Department of Special Needs Education, Oita University, 700 Dannoharu, Oita, Japan; 3 Graduate School of Human Sciences, Osaka University, 1-2 Yamadaoka, Suita, Japan; 4 Division of Child Neurology, National Hospital Organization Osaka Toneyama Medical Center, 5-1-1 Toneyama, Toyonaka, Japan; 5 Department of Neurology, National Hospital Organization Osaka Toneyama Medical Center, 5-1-1 Toneyama, Toyonaka, Japan; 6 Department of Clinical Laboratory and Biomedical Sciences, Osaka University Graduate School of Medicine, 1-7 Yamadaoka, Suita, Japan; 7 Department of Neurology, Osaka University Graduate School of Medicine, 2-2 Yamadaoka, Suita, Japan; 8 Department of Neurology, National Hospital Organization Aomori National Hospital, 155-1 Megasawa-Hirano, Aomori, Japan; 9 Department of Neurology, Yokohama Rosai Hospital, 3211 Kozukue, Yokohama, Japan; 10 Faculty of Social Sciences, Nara University, 1500 Misasagi, Nara, Japan

**Keywords:** Myotonic dystrophy, Neuromuscular diseases, Patient-reported outcome measures, Quality of life, Symptom impact

## Abstract

**Background:**

Although functional impairment in patients with myotonic dystrophy is an important determinant of the quality of life (QoL), patients’ subjective evaluation of their symptoms may also affect their QoL. The aim of this study was to investigate the association between subjective symptom impact and the QoL of patients with myotonic dystrophy, after controlling for functional impairment.

**Methods:**

Eligible patients with myotonic dystrophy type 1 (DM1) were recruited from four hospitals in Japan. The subjective symptom impact of four symptoms (muscle weakness, fatigue, pain, and myotonia) and overall QoL were evaluated using the Individualized Neuromuscular Quality of Life (INQoL) questionnaire. Functional impairment was assessed using the modified Rankin Scale.

**Results:**

Seventy-seven patients with DM1 were included in this study. Overall QoL was significantly associated with subjective symptom impact of muscular weakness, fatigue, pain, myotonia, swallowing difficulty, and droopy eyelids. In the regression models, disease duration (beta = 0.11) and moderate to severe functional impairment (beta = 0.33) explained a significant part of the overall QoL. Furthermore, muscular weakness, fatigue, and myotonia significantly explained additional variance of the overall QoL (beta = 0.17–0.43).

**Conclusions:**

Subjective symptom impact and functional impairment are independent features influencing the QoL of Japanese patients with DM1.

## Background

Myotonic dystrophy type 1 (DM1) is the most common muscular dystrophy in adults, and is characterized by various symptoms, including progressive muscular weakness, fatigue, and myotonia [1]. These symptoms often affect patients’ lives, including daily activities and social participation, which in turn negatively affect the quality of life (QoL) [2, 3]. Generally, symptom severity is considered the strongest predictor of patients’ QoL [4]. Functional impairment is one of the major factors that affect activities of daily living and social participation in neuromuscular disorders; however, cognitive, psychosocial, and subjective factors also strongly contribute to QoL [5–8]. As illustrated by the disability paradox, patients with severe disability do not necessarily experience worse QoL, which highlights the influence of one’s personal disability experience [9, 10]. Such phenomenon suggests that subjective experience of a disorder, including estimation of symptoms by patients and psychological adjustment, may be an essential factor of their QoL [11]. Most validated QoL measures, including the 36-Item Short Form Health Survey (SF-36), are generic measures used in people with various conditions [12]. Whereas generic measures are useful to evaluate QoL in comparison to general population, disease-specific measures (e.g., Individualized Neuromuscular Quality of Life [INQoL] questionnaire) have advantages to capture experience in individuals with muscular dystrophies [2, 13].

Recently, patient-reported outcomes have increasingly been considered important in clinical trials [2, 14]. Although the symptom severity has been well studied for neuromuscular disorders, the impact of patients’ subjective assessment of difficulties in activities and participation and the perceived importance of these difficulties on their QoL has not been well investigated. Furthermore, the assessment of symptoms by a clinician and subjective evaluation of symptoms by patients may differ [15]. Therefore, patients’ QoL could be affected by their subjective evaluation of symptom severity, limitations in activities, and restrictions on participation associated with muscular diseases [16, 17]. Consequently, even for patients experiencing symptoms of similar severity, QoL may differ depending on the restrictions experienced and the perceived importance of these activities and participation. In the current study, we tested the hypothesis that subjective symptom impact will contribute to QoL even after controlling for functional impairment in Japanese patients with DM1.

## Methods

### Participants

Patients were recruited from four hospitals in Japan (Osaka Toneyama Medical Center, Aomori National Hospital, Osaka University Hospital, and Yokohama Rosai Hospital). The eligibility criteria for patients were a genetic diagnosis of DM1, being aged ≥18 years, and provision of informed consent after the procedures had been fully explained. Since the data were collected as part of a larger study, some of the data included in the analysis overlapped with those from our previous study [18]. The CTG repeat length of the patients, measured by several commercial laboratories, was obtained from the hospitals’ electronic medical records. The laboratories employed Southern blot hybridization of restriction-enzyme-digested genomic DNA that was extracted from the patients’ blood. Therefore, the modal allele or the median of the smear range was used in statistical analyses.

### Ethics, consent, and permissions

Informed consent was obtained from all patients who participated in this study. This study was conducted in accordance with the World Medical Association’s Declaration of Helsinki and was approved by the Osaka University Clinical Research Review Committee (no. 14480).

### Measures

#### Subjective symptom impact and QoL

The subjective burden of symptoms and QoL were measured using the Japanese version of the Individualized Neuromuscular Quality of Life (INQoL) [18, 19]. The INQoL was developed through qualitative and quantitative studies of neuromuscular diseases, including DM1. The measure includes common symptoms of neuromuscular diseases, i.e., muscular weakness, pain, fatigue, myotonia, droopy eyelids, double vision, and swallowing difficulty. Each symptom is evaluated on three scales: severity of the symptom, difficulties caused by the symptom, and the perceived importance of the difficulties caused by the symptom. Symptom scores range from 0 to 100, and indicate subjective impact of each symptoms. A higher score indicates greater symptom impact.

The QoL index consisted of the life domain scales of the INQoL, which also includes independence, social relationships, emotions, and body image, apart from symptom scales. The subjective symptoms and life domain scores are independent indices. Thus, the QoL index represents a patient’s overall QoL on a scale from 0 to 100, which was calculated by the above four QoL aspects. A higher score is indicative of worse QoL.

#### Functional impairment

We assessed functional impairment using the modified Rankin Scale (mRS) [20], which has previously been used as an index of functional impairment in neuromuscular disorders [18, 21, 22]. The functional impairment was evaluated by each patient’s primary physician, with scores on the mRS ranging from 0 (no symptoms) to 5 (severe disability).

### Statistical analysis

Statistical analyses were performed using R 3.4.1 statistical software (R Core Team, Vienna, Austria). Associations between subjective symptom impact and QoL were evaluated using Spearman rank correlation coefficient because the symptom scores were positively skewed.

Based on the existing studies [23, 24], we included the four symptoms measured by the INQoL, i.e., weakness, fatigue, pain, and myotonia, in the regression models. The two-step model was tested with Gaussian distribution of the response variable (overall QoL) and identity link function. Step 1 was tested with demographic (sex, age, and years of education) and clinical variables (disease duration, the number of the CTG repeats, and functional impairment) as explanatory variables. Step 2 was tested with step 1 and four subjective symptom measures as explanatory variables. In addition, we examined another model including all seven symptoms for exploratory purpose. The mRS score was dichotomized to mild functional impairment (0) for scores 0–2 and moderate to severe functional impairment (1) for scores of 3–5 based on a previous study [21] and included as a dummy-coded variable. We used the Q-Q plot to confirm the assumption that the residuals of the regression were normally distributed. In the multiple regression analysis, we also calculated the variance inflation factor (VIF) to assess potential multicollinearity between predictor variables, where a VIF > 10 was indicative of multicollinearity between predictors, and a VIF of 5–10 suggested a multicollinearity problem. The model’s goodness-of-fit was measured using Nagelkerke’s pseudo R^2^ and Akaike information criterion (AIC) of the generalized linear models. The significance level was set at a two-tailed *p* < 0.05.

## Results

Seventy-seven patients with genetically confirmed DM1 were included in this study (Table 1). The overall QoL was significantly correlated to patients’ subjective symptom impact, except for double vision (*p* = 0.062) (Table 2). Functional impairment was also moderately correlated with QoL (*rho* = 0.51 [95% CI: 0.32, 0.66], *p* < 0.001).

**Table 1:** Demographic and clinical variables of the patients

	Mean (SD) or Number [%]
Sex (male/female)	36/41 [female 53.2]
Age	47.3 (10.5)
Years of education	13.5 (1.8)
Onset age	29.7 (13.1)
Disease duration (years)	17.5 (11.1)
Number of CTG repeats^a)^	689.8 (447.6)
Employment	29 [37.7]
Symptom impact	
Weakness	54.1 (26.0)
Pain	22.7 (27.9)
Fatigue	46.6 (27.4)
Myotonia	36.3 (30.8)
Droopy eyelids	12.6 (24.0)
Double vision	12.6 (21.9)
Swallowing difficulty	29.4 (26.2)
Overall QoL	46.4 (23.0)

^a)^ estimated using Southern blot

*QoL* quality of life

**Table 2:** Spearman’s correlation coefficients (95% CI) between subjective symptom impact and overall QoL

	Pain	Fatigue	Myotonia	Droopy eyelids	Double vision	Swallowing difficulty	Overall QoL
Weakness	0.43^***^	0.66^***^	0.46^***^	0.36^**^	0.16	0.41^***^	0.86^***^
	(0.23, 0.60)	(0.51, 0.77)	(0.27, 0.62)	(0.15, 0.54)	(−0.06, 0.37)	(0.20, 0.58)	(0.78, 0.91)
Pain		0.63^***^	0.51^***^	0.33^**^	0.32^**^	0.32^**^	0.58^***^
		(0.48, 0.75)	(0.32, 0.66)	(0.12, 0.52)	(0.11, 0.51)	(0.11, 0.51)	(0.41, 0.71)
Fatigue			0.53^***^	0.30^**^	0.23^*^	0.42^***^	0.75^***^
			(0.35, 0.68)	(0.08, 0.49)	(0.01, 0.43)	(0.21, 0.59)	(0.63, 0.83)
Myotonia				0.25^*^	0.31^**^	0.47^***^	0.58^***^
				(0.03, 0.45)	(0.10, 0.50)	(0.27, 0.62)	(0.41, 0.71)
Droopy eyelids					0.42^***^	0.27^*^	0.33^**^
					(0.22, 0.59)	(0.05, 0.47)	(0.12, 0.52)
Double vision						0.23^*^	0.21
						(0.01, 0.44)	(−0.01, 0.42)
Swallowing difficulty							0.39^***^
							(0.19, 0.57)

^***^: *p* < 0.001, ^**^: *p* < 0.01, ^*^: *p* < 0.05

*CI* confidence interval, *QoL* quality of life

Demographic and clinical variables were entered into Model 1, and overall QoL was significantly explained by disease duration and functional impairment (Table 3). Longer disease duration and moderate to severe functional impairment are associated with lower QoL (Table 3). The subjective symptom impact was then added as an explanatory variable (Model 2), and the subjective impact of weakness, fatigue and myotonia explained a significant proportion of the variance in overall QoL, even after controlling for demographic variables and functional impairment (Table 3). Higher subjective symptom impacts of weakness, fatigue, and myotonia were associated with lower QoL. The Nagelkerke’s pseudo *R*^2^ that reflects explained variance in the overall QoL was high in Model 2. The AIC also supported the better fit of the model. VIF values for multicollinearity were not greater than the threshold in both Models 1 and 2 (VIF < 3.0).

**Table 3:** Associations between explanatory variables and overall QoL in DM1

Explanatory variable	Model 1^a^		Model 2^b^	
	Coefficient (95% CI)	*P*-value	Coefficient (95% CI)	*P*-value
Demographic variables and functional impairment				
Sex	−0.11 (− 0.47, 0.26)	0.570	0.02 (− 0.18, 0.23)	0.834
Age	0.08 (−0.14, 0.30)	0.485	−0.02 (− 0.15, 0.10)	0.722
Years of education	0.05 (−0.16, 0.26)	0.616	0.00 (−0.11, 0.12)	0.982
Disease duration	0.40 (0.20, 0.59)	< 0.001	0.12 (0.00, 0.23)	0.047
Number of CTG repeats	−0.08 (−0.29, 0.12)	0.421	−0.06 (− 0.17, 0.05)	0.317
Functional impairment	0.82 (0.40, 1.23)	< 0.001	0.31 (0.07, 0.56)	0.014
Subjective symptom impact				
Weakness			0.47 (0.30, 0.64)	< 0.001
Pain			0.09 (−0.05, 0.23)	0.228
Fatigue			0.22 (0.06, 0.38)	0.009
Myotonia			0.16 (0.02, 0.29)	0.029

^a^: Nagelkerke’s pseudo *R*^*2*^ = 0.41, AIC = 195.7

^b^: Nagelkerke’s pseudo *R*^*2*^ = 0.84, AIC = 105.4

*CI* confidence interval, *AIC* Akaike information criterion, *QoL* quality of life

When we included all seven symptoms of the INQoL for exploratory purpose (i.e., weakness, pain, fatigue, myotonia, droopy eyelids, double vision, and swallowing difficulty), the significant explanatory variables were the same as shown in Table 3. The remaining three symptom scores did not significantly explain the additional variance of QoL when we added all subjective symptom variables to Model 2 (droopy eyelids: *p* = 0.43; double vision: *p* = 0.68; swallowing difficulty: *p* = 0.79).

## Discussion

The subjective symptom impact explained a significant proportion of the overall QoL among patients with DM1, after controlling for demographic variables and functional impairment. Our findings are consistent with those of previous studies, which reported the association of disease duration and functional impairment with QoL [25–27]; however, the relative contribution of these variables was limited when subjective symptom impact was explained. In fact, a substantial variance in QoL was explained by patients’ subjective evaluation of the burden caused by muscular weakness, fatigue, and myotonia. Of these subjective factors, muscle weakness was the strongest predictor of QoL among the symptoms examined in this study.

Most subjective symptom impacts were moderately to strongly associated with overall QoL measured by the INQoL. Most activities of daily living were strongly affected by muscle weakness in patients with neuromuscular diseases, which naturally worsens QoL, and fatigue and myotonia were associated with social participation and daily activities [28]. In contrast, the correlation between double vision and QoL was weak and not significant in this study. This may be because it was the least frequent symptom among those evaluated in the INQoL, consistent with a previous report [23].

Because QoL is a subjective phenomenon, patients’ evaluation of symptoms constitutes an important part of QoL, in addition to the objective assessment of disease severity [2, 3]. Further, the subjective evaluation of the symptom impact may also differ by symptoms [28]. The regression models showed that subjective symptom impact of muscular weakness, fatigue, and myotonia explained additional variance in QoL even after controlling for demographic and clinical variables and functional impairment. The results showed that the subjective symptom impact of these domains uniquely contributed to patients’ QoL, suggesting the relative importance of these symptoms in understanding QoL in patients with DM1. Thus, reducing the burden of these symptoms is an important target for interventions to improve QoL in patients with myotonic dystrophy. In contrast to previous studies [23, 29], here, the CTG repeat length was not associated with overall QoL and symptoms. The discrepancy in the results may be caused by differences in the methods used to measure the repeat length and ambiguity in the CTG repeat length measurements caused by somatic mosaicism. In addition, the age-related expansion of the repeat length, which is due to somatic instability confounding with patient’s age, also influence the estimation of the measurements. Such characteristics may weaken the relationship between CTG repeat and overall QoL in the current study.

Our study suggests the significant effects of subjective symptom impact on QoL among patients with DM1, which might account for the disparity between objective disease severity progression and QoL that has been reported in a few longitudinal studies [11, 30, 31]. It is possible that QoL and symptom impact could be influenced by a response shift phenomenon, which is defined as a change in internal standards [3, 32]. Patients’ perceived impact of a disease is an important mediator of QoL, which may explain the noted variation in QoL among patients with symptoms of similar severity. In fact, disease perception is one of the determinants of psychological distress and/or coping [7, 33]. Therefore, although definitive evidence for the effectiveness of psychosocial interventions to improve disease perception in patients with muscular diseases is unavailable [34], it is plausible that psychosocial interventions could optimize QoL in these patients [35, 36]. A large international study demonstrated that cognitive behavior therapy for patients with myotonic dystrophy improved physical activity and participation, but it did not improve their QoL and disease burden [37]. Although such results were affected by the fact that the intervention did not focus on subjective disease burden and QoL, more direct interventions may be needed [35].

In summary, our findings indicate that subjective symptom impact and functional impairment are independent features associated with QoL among Japanese patients with DM1.

### Limitations

The limitations of our study should be acknowledged. First, although the INQoL covers the major components of QoL for patients with DM1, other contributing factors could affect QoL. Recently, the Myotonic Dystrophy Health Index, which measures the burden of disease in myotonic dystrophy, has been validated in several languages [36, 38–40]. The INQoL is a measure of how disease symptoms affect the patient’s perspective of their disease burden [18, 19]. Thus, combining the two instruments may be desirable to determine patients’ subjective experiences of the disease better. Second, we could not examine environmental factors, such as social and welfare support, that could affect patients’ QoL; these factors are known to affect patients’ QoL [6]. Third, cognitive impairment, disease awareness, and apathy, which are associated with disease severity, may have partially moderated the associations between subjective evaluation of disease impact and QoL [15, 41, 42]. Lastly, the QoL measures developed using item response theory or Rasch models, such as Quality of Life in Genetic Neuromuscular Disease Questionnaire [43, 44], would be more appropriate to obtain precise estimates for these associations.

## Data Availability

The datasets generated and/or analyzed during the current study are not publicly available due to privacy constraints relating to the ethical approval but are available from the corresponding author on reasonable request.

## Abbreviations

DM1: myotonic dystrophy type 1
INQoL: Individualized Neuromuscular Quality of Life
mRS: modified Rankin scale
QoL: Quality of life
